# Costs and Benefits of Native Language Similarity for Non-native Word Learning

**DOI:** 10.3389/fpsyg.2021.651506

**Published:** 2021-05-28

**Authors:** Viorica Marian, James Bartolotti, Aimee van den Berg, Sayuri Hayakawa

**Affiliations:** ^1^Department of Communication Sciences and Disorders, Northwestern University, Evanston, IL, United States; ^2^Life Span Institute, University of Kansas, Lawrence, KS, United States

**Keywords:** language learning, cross-language similarity, second language, language acquisition, vocabulary learning

## Abstract

The present study examined the costs and benefits of native language similarity for non-native vocabulary learning. Because learning a second language (L2) is difficult, many learners start with easy words that look like their native language (L1) to jumpstart their vocabulary. However, this approach may not be the most effective strategy in the long-term, compared to introducing difficult L2 vocabulary early on. We examined how L1 orthographic typicality affects pattern learning of novel vocabulary by teaching English monolinguals either Englishlike or Non-Englishlike pseudowords that contained repeated orthographic patterns. We found that overall, the first words that individuals learned during initial acquisition influenced which words they acquired later. Specifically, learning a new word in one session made it easier to acquire an orthographically similar word in the next session. Similarity among non-native words interacted with native language similarity, so that words that looked more like English were easier to learn at first, but they were less effective at influencing later word learning. This demonstrates that although native language similarity has a beneficial effect early on, it may reduce learners' ability to benefit from non-native word patterns during continued acquisition. This surprising finding demonstrates that making learning easier may not be the most effective long-term strategy. Learning difficult vocabulary teaches the learner what makes non-native words unique, and this general wordform knowledge may be more valuable than the words themselves. We conclude that native language similarity modulates new vocabulary acquisition and that difficulties during learning are not always to be avoided, as additional effort early on can pay later dividends.

## Introduction

Children often excel at learning new languages—consider international adoptees who rapidly acquire their “second first language” (Roberts et al., 2005)—whereas for adults, learning a second language (L2) has traditionally been thought to be a more difficult task (Liskin-Gasparro, 1982). There is now substantial evidence that, for children and adults alike, the ability to successfully learn a second language can be moderated by complex interactions between contextual, sociocultural, cognitive, and affective variables (see Dixon et al., 2012 and Ortega, 2013 for reviews), as well as characteristics of the first (L1) and second (L2) languages. Oftentimes, learners can take advantage of similarities between the L1 and L2 by relying on existing skills and knowledge to learn the new language (i.e., cross-linguistic transfer or cross-linguistic influence; Ringbom, 2007; Jarvis and Pavlenko, 2008). Other times, transfer from the L1 can inhibit learning, such as when L1 knowledge is inappropriately applied (Laufer, 1988; Eckman, 2004) or when it interferes with the acquisition of L2-specific representations (Flege, 1987; Goldrick et al., 2014). Seeing language acquisition as an incremental process (that successively builds on previously-learned information), the costs and benefits of cross-linguistic influences at early stages of learning could have cascading consequences for later acquisition. In the current study, we examine the developmental trajectory of cross-linguistic influences on novel word learning and the role of orthographic similarity to previously-learned native and non-native words.

### Effects of Cross-Linguistic Transfer on New Word Learning

Native language similarity has long been known to be a powerful resource for language learning. In many cases, the ease and efficiency of language acquisition can be modulated by existing language knowledge and the actual (Ringbom, 2007) or perceived (Kellerman, 1978; Odlin, 1989) formal similarities between languages. Similarities between languages and cross-linguistic transfer can be found at multiple levels of representation, such as phonology (e.g., Melby-Lervåg and Lervåg, 2011; Wrembel, 2011), orthography (e.g., De Groot and Keijzer, 2000; Ellis, 2008), and morphology (e.g., Hancin-Bhatt and Nagy, 1994; Ecke, 2015). During vocabulary learning, cognates, which overlap across languages in both orthographic form and meaning, are often more readily acquired than non-cognates (Lotto and de Groot, 1998; De Groot and Keijzer, 2000). In addition to allowing learners to draw from existing knowledge, L1-L2 similarity may facilitate integration of novel wordforms into the existing lexico-semantic network (Shirai, 1992; MacWhinney, 1997; Comesaña et al., 2009; De Groot, 2011), which can result in more robust encoding during early stages of acquisition (Ellis and Beaton, 1993), as well as more fluid retrieval at higher levels of proficiency (e.g., Comesaña et al., 2012).

SLA studies conducted in more naturalistic contexts (e.g., classrooms) have also found advantages for learning cognates (e.g., Cunningham and Graham, 2000; Tonzar et al., 2009; Vidal, 2011; Otwinowska and Szewczyk, 2019; Puimège and Peters, 2019), but have produced more mixed results (e.g., Rogers et al., 2015; Otwinowska et al., 2020; see Otwinowska, 2015 for review). Some evidence suggests that cognate facilitation may be contingent on formal training in cognate recognition (Tréville, 1996; Dressler et al., 2011), suggesting that learners may not always be aware of formal similarities. Indeed, using contrastive analysis to highlight similarities and differences between the L1 and L2 can be highly effective (Laufer and Girsai, 2008; Lin, 2015; Helms-Park and Perhan, 2016), and students tend to respond positively to this type of language instruction (Brooks-Lewis, 2009). Additionally, the trade-off between ecological validity and control over stimulus characteristics (e.g., word frequency, orthographic overlap) may also contribute to the more tenuous cognate effects found in SLA studies compared to laboratory experiments (but see Otwinowska and Szewczyk, 2019 for an exception and Otwinowska, 2015 for discussion).

Psycholinguistic studies using carefully controlled real or artificial word stimuli have revealed that the cognate advantage increases with the degree of orthographic overlap (De Groot, 2011; Comesaña et al., 2015), and that even without semantic overlap, vocabulary acquisition can be facilitated for novel words that are orthographically (De Groot, 2011; Bartolotti and Marian, 2014, 2017; Marecka et al., 2021) or phonologically (Ellis and Beaton, 1993; Service and Craik, 1993; Roodenrys and Hinton, 2002; Storkel and Maekawa, 2005; Storkel et al., 2006) similar to L1 words. For instance, Meade et al. (2018) observed that pseudowords with a higher number of L1 orthographic neighbors were produced more accurately than low-density words. Like cognates, the advantage for words that resemble L1 wordforms could result from more effective use of, and integration with, existing lexical and semantic knowledge. This can be done explicitly through association-based strategies (e.g., mneumonic methods; Atkinson and Raugh, 1975; Meara, 1980; Paivio and Desrochers, 1981; see Hulstijn, 1997 and Nation, 1982 for reviews) or implicitly through the co-activation of orthographically or phonologically similar L1 words and associated meanings (Holcomb et al., 2002; Van Hell and Tanner, 2012).

Words with familiar orthographic or phonological features may additionally benefit word learning by allowing learners to exploit knowledge of sublexical regularities in the L1. For instance, Bartolotti and Marian (2014) found that vocabulary acquisition is facilitated when pseudowords are designed to reuse native language letter patterns (i.e., higher bigram probabilities; see also Bartolotti and Marian, 2017). Phonological overlap between the L2 and L1 can additionally facilitate learning by increasing pronounceability (Ellis and Beaton, 1993; Service and Craik, 1993), which could enable learners to rely on phonological knowledge stored in long-term memory (Cheung, 1996; Storkel and Maekawa, 2005; De Groot, 2011) and the mental rehearsal of novel phonological forms (Papagno et al., 1991; Ellis and Sinclair, 1996).

Despite potential advantages, lexical and sublexical similarities between languages can also introduce costs when they are over-applied or block acquisition of new features during learning. For example, a German learner of English may say, “I need a loffel for my soup,” under the mistaken belief that the German word *Löffel* (meaning spoon) is an English cognate (Eckman, 2004). This type of confusion can be especially likely when a novel word overlaps with a known word in some respects, but not others (i.e., “deceptive transparency;” Laufer, 1988), such as when an L2 word overlaps in form but not meaning with an L1 word (i.e., false friends; e.g., the German word *Rat*, which means “advice”). A different problem occurs when similarity to the L1 interferes with acquisition of L2-specific features or regularities, as seen with spoken accents. Sounds in the L2 that are similar to an existing L1 sound are actually *more* difficult to pronounce accurately than completely new sounds (Flege, 1987). Even speakers who have mastered L2 phonology still pronounce cognate words with more of an accent than non-cognates, due to cognates' high L1 similarity (Amengual, 2012, 2016; Goldrick et al., 2014; consistent with Anderson's (1983) *transfer to somewhere* principle).

Kaushanskaya and Marian (2009) found that, compared to when phonologically atypical pseudowords were presented alone, learning was impaired when words were presented bimodally with *typical* L1 orthographic forms. Such effects could be explained by the increased activation of L1 representations in response to familiar wordforms, which could compete with the more recently acquired L2 representation. For instance, psycholinguistic studies have demonstrated that visual word recognition can be inhibited by orthographically-related primes (both within and across languages; e.g., Bijeljac-Babic et al., 1997) that can compete for selection. Cross-language lexical activation can be reduced, however, by priming different script bilinguals (e.g., Hindi-English) with a particular writing system (Dubey et al., 2018), as well as by priming same script bilinguals with language-specific sublexical cues (e.g., bigrams) that are uncommon or orthotactically illegal in one language but not the other (Casaponsa et al., 2020). Similarly, language discrimination is facilitated by orthographic markers that signal language membership (Casaponsa et al., 2014; Oganian et al., 2015). These patterns of facilitation and interference indicate a high degree of cross-linguistic interactivity within the language system, which can play a significant role in vocabulary acquisition and be amplified by similarities at the lexical and sublexical level of processing.

### Effects of Within-Language Transfer on New Word Learning

Similarity to the L1 can yield significant benefits during early stages of word learning by encouraging cross-linguistic transfer. The recognition and use of L2-specific patterns, however, is key to long-term success in developing L2 vocabulary. Adults who had completed 1 year of university-level Spanish courses were able to learn new words with a large number of Spanish neighbors (i.e., words that differed from many Spanish words by only a single phoneme) at a higher rate than words with a low number of Spanish neighbors (Stamer and Vitevitch, 2012). This ability to learn words with more L2 neighbors provides evidence that similarities *within* an L2 benefit learning. The application of within-language knowledge for novel word learning can additionally vary as a function of individual differences such as L2 proficiency (e.g., Horst et al., 1998; Zahar et al., 2001; Pulido, 2003; Tekmen and Daloglu, 2006; Ma et al., 2015; Otwinowska and Szewczyk, 2019). For instance, Ma et al. (2015) observed that L2 proficiency was positively associated with learners' ability to learn the meanings of novel pseudowords embedded in sentences. Studies employing incidental learning paradigms have similarly observed that higher L2 proficiency and larger L2 vocabulary sizes facilitate novel vocabulary acquisition during reading (e.g., Horst et al., 1998; Tekmen and Daloglu, 2006). Such findings suggest that as proficiency in the L2 increases, so too does the strength of within-L2 facilitation, creating a positive feedback loop where L2 word learning becomes easier as L2 vocabulary size increases. Proficiency can also modulate the contribution of other domain-general cognitive abilities (Cheung, 1996; Gathercole and Masoura, 2005). For instance, Cheung (1996) found that greater short-term memory capacity was associated with better L2 vocabulary learning for individuals with low, but not high L2 proficiency (but see Majerus et al., 2008 who found independent effects of STM and L2 phonological knowledge). Bartolotti et al. (2011) observed that inhibitory control and bilingual experience independently predicted how well learners were able to extract statistical regularities of word boundaries in an artificial Morse Code language after listening to another language with conflicting patterns. Bilingual experience facilitated word learning when interference was low, whereas inhibitory control predicted performance when interference was high (see also Wang and Saffran, 2014, who observed a bilingual advantage for detecting regularities in an artificial tonal language). The ability to extract and apply regularities within the L2 can therefore vary depending on both individual differences in cognitive and linguistic abilities, as well as characteristics of the learning task.

Though gains are likely to compound with increased L2 experience, the beneficial effect of within-L2 similarity applies even at the earliest stages of acquisition (McLaughlin et al., 2004; Bartolotti and Marian, 2017). After only 14 h of classroom study, novice L2 learners' neural responses indicated familiarity with words they had seen before, even though behaviorally they only identified words at chance performance (McLaughlin et al., 2004; Osterhout et al., 2006). After only one session of training in an artificial language, learners demonstrate that they have learned letters' relative frequencies in the language, and can use this information to fill gaps in their knowledge of the new language (Bartolotti and Marian, 2017). Other statistical regularities governing word boundaries can be learned from continuous speech after as little as 20 min of exposure (Saffran et al., 1999; Karuza et al., 2013), and this knowledge of word boundaries can directly influence subsequent vocabulary acquisition (Mirman et al., 2008). Together, these findings demonstrate that learners are able to extract L2 regularities based on even brief amounts of exposure, which can then be used to support further learning.

### Effects of Cross-Linguistic Influence on Within-Language Transfer

While there has been substantial research investigating the independent effects of between- and within-language transfer on vocabulary acquisition, relatively less is known about their potential interactions—specifically, whether native language orthographic similarity modulates transfer between non-native words during subsequent learning. A significant body of research has shown that the strategies individuals use to process words within the L1 is influenced by their orthographic system (e.g., Hakuta, 1982; MacWhinney and Bates, 1989), and that these same processes may be used to decode words in an L2 (e.g., Koda, 1998; Mori, 1998; Hamada and Koda, 2008). As a result, sensitivity to L2-specific orthotactics can vary as a function of similarity between L1 and L2 orthographic systems. For instance, Koda (1998) observed that ESL learners with L1 Korean (who utilize a syllable-based writing system, *hangul*) were more sensitive to English orthotactic violations (i.e., illegal letter sequences) than L1 Chinese speakers (who utilize a morpheme-based logographic writing system). The author conjectures that the Korean speakers' increased sensitivity to L2 intraword structures likely results from their greater need to attend to component letters and sequences during L1 decoding relative to Chinese speakers. This finding suggests that L1 experience can modulate learning of L2-specific regularities, with variable outcomes depending on how well the strategies acquired for the L1 can be applied to the L2 (see also Koda, 1990, 1993 for similar effects of L1 orthography and transfer on L2 reading comprehension strategies and Koda and Zehler, 2008, for review). The present study examines the possibility that effects of cross-linguistic similarity may be observed when languages overlap, not only in their orthographic systems as a whole, but in the orthographic forms of particular words.

Preliminary support for this possibility comes from another study by Koda (1989), who found that Japanese L2 learners whose L1 (Korean or Chinese) overlapped with one type of Japanese script (*kanji*, a logographic, meaning-based system), but not another (*hiragana*, a phonetic lettering system), outperformed learners with orthographically dissimilar L1s in learning vocabulary of both scripts. Furthermore, initial L1-similarity advantages for word learning compounded to yield later benefits for more complex tasks, such as reading comprehension. While these findings indicate that L1 similarity for a subset of L2 vocabulary can facilitate the acquisition of other L2-specific wordforms (potentially via transfer of phonological representations that map to both *kanji* and *hiragana*), learners with knowledge of logographic characters could have benefited from overlap in both orthographic form and meaning (akin to cognate facilitation). The present study therefore examines whether similar benefits of cross-linguistic influence on within-language transfer can be observed when a subset of novel words overlap with the L1 in sublexical properties alone.

Given that adult language learners' primary approach when they start learning a new language is typically to identify and reuse perceived similarities to their native language (Ringbom and Jarvis, 2011), it would be consequential to know how increased activation and use of L1 knowledge during early stages of learning affects learners' ability to later rely on regularities within the L2. One possibility is that identifying useful similarities between the L1 and L2 during initial acquisition will enhance the ability to learn and use similarities within the L2. For instance, learning L2 words that share orthographic features with the L1 could establish a stronger base of knowledge to be used as exemplars for subsequently learned words with L2-specific features. In addition to potential differences in the strength of L2 (exemplar) representations, the cognitive processes and strategies engaged while learning words that resemble the L1 could increase the salience and use of L2 regularities.

There are also reasons to expect that transfer between L2 words could instead be facilitated by the initial acquisition of wordforms that are *dissimilar* to the L1. For instance, the greater challenges associated with learning dissimilar words could serve as a form of desirable difficulty (Bjork and Bjork, 2011), which could elicit higher levels of involvement (Craik and Lockhart, 1972; Laufer and Hulstijn, 2001; Rice and Tokowicz, 2020) or motivation (Dörnyei and Ushioda, 2009; Dörnyei, 2019), resulting in deeper processing and greater sensitivity to L2-specific patterns. It may also be the case that words with less typical L1 orthography would elicit relatively less activation of L1 representations that could interfere with the identification and use of L2 features (e.g., Amengual, 2012, 2016; Goldrick et al., 2014). If so, we may observe greater within-L2 transfer after learning words with orthographic features that are uncommon in the L1.

In the present study, we introduce the concept of “bridge” words as a means to investigate cross-linguistic influences on transfer between novel vocabulary and the potential utility of bridge words for teaching learners about useful features of non-native words. Bridge words are defined as novel words that contain letter sequences that are common among the non-native vocabulary to facilitate subsequent learning. Acquiring a bridge word (e.g., *haner* in the current study) may make it easier to learn a similarly spelled “terminus” word (e.g., *hajer*) to which it is connected because of orthographic feature overlap. Some bridge words use letter sequences that are also common in the L1, which may make them easier to acquire, whereas other bridge words have orthographic forms that are uncommon in the L1. To examine the effect of L1 similarity on bridge words' utility, we designed contrasting sets of pseudowords and taught participants one of two word lists across two sessions. Participants were first taught bridge words comprised of letter sequences (i.e., bigrams) that were either typical (i.e., “Familiar;” e.g., *haner*, meaning “bride”) or atypical of English words (i.e., “Unfamiliar;” e.g., *vobaf*, meaning “cloud”), followed by an immediate test where they produced the new word when cued with its meaning. Two weeks later, participants returned to learn terminus words that were related to their previously-learned bridge words (e.g., *hajer, tobaf*), and were again tested immediately. If we observe a general benefit for terminus word acquisition based on bridge word knowledge, it would suggest that learners are able to use orthographic similarities within the non-native vocabulary to facilitate subsequent learning. Critically, if we observe different effects of bridge words in the Familiar and Unfamiliar conditions, it would suggest that native language orthotactic typicality can modulate how knowledge specific to non-native words is used. Native language similarity may improve bridge-to-terminus transfer, by accentuating word-to-word similarity as a learning tool, or it may interfere, by hindering acquisition of non-native patterns.

## Methods

### Participants

A power analysis to determine sample size was run with Monte Carlo simulations using the SIMR package in R for use with linear mixed effect models (Green and Macleod, 2016). An effect size for the influence of L1 orthographic bigram typicality on learning was obtained from word learning data in Bartolotti and Marian (2017), providing a fixed effect estimate of 10% on learning accuracy. Population mean and variance were obtained from pilot data. Power estimates were calculated for simulated sample sizes from 20 to 40. Power >0.8 was obtained with 30 participants, and power >0.9 was obtained with 38 participants.

Sixty-five English-speaking adults initially participated after providing informed consent in accordance with the university's institutional review board, and were randomly assigned to learn Familiar or Unfamiliar word lists. Participants' language profiles were collected using the LEAP-Q (Marian et al., 2007). Non-verbal IQ was assessed using the matrix reasoning subtest of the Wechsler Abbreviated Scale of Intelligence (PsychCorp, 1999). Verbal memory was assessed using the verbal paired associates test of the Wechsler Memory Scale III (Wechsler, 1997).

As the novel vocabulary used in the present study was controlled for orthographic wordform similarity to English (i.e., bigram and biphone probability), but not other languages, only native English-speakers with minimal second language knowledge were included in the final sample. Eligible participants had self-reported second language proficiencies (speaking, listening, and reading composite score) of less than 3 (corresponding to “low” proficiency) on a scale of 0–10 (ranging from “none” to “perfect”). This language knowledge criterion was applied prior to data analysis and excluded all bilinguals and multilinguals (*N* = 27), yielding a final sample size of 38 participants (Familiar group *N* = 17, Unfamiliar group *N* = 21). Participants in the Familiar and Unfamiliar groups did not differ in non-verbal IQ standard scores (Familiar *M* = 111.0, *SE* = 0.55, Unfamiliar *M* = 110.5, *SE* = 0.38, *t*(28.1) = 0.17, n.s.) or verbal memory standard scores (Familiar *M* = 13.0, *SE* = 0.15, Unfamiliar *M* = 13.4, *SE* = 0.13, *t*(32.6) = 0.45, n.s.). All participants were students at Northwestern University who completed the study in a classroom-like setting in exchange for extra credit.

### Materials

The Familiar and Unfamiliar word lists each contained 48 five-letter words with alternating consonants and vowels in CVCVC format (Q, Y, and X were not used in either language). Two versions of each word list were created, one per training session. Vocabulary items in the first list were used to examine L1 influences on learning, and were selected by evaluating 10,000 randomly generated non-words for English similarity. Though word lists were presented visually, psycholinguistic evidence suggests that phonological forms of words are co-activated even in response to unimodal orthographic inputs (e.g., Perfetti and Bell, 1991; Ferrand and Grainger, 1993; Grainger and Ferrand, 1994; Brysbaert et al., 1999; Van Wijnendaele and Brysbaert, 2002; Brysbaert and Van Wijnendaele, 2003; Grainger et al., 2006; Braun et al., 2009). English similarity was therefore determined based on both bigram and biphone probabilities. Phonological forms of each novel word were determined using the eSpeak speech synthesizer software, version 1.48.15 for Linux (Duddington, 2012). Pronunciations were obtained as IPA transcriptions using eSpeak's EN-US American English voice, and were translated from IPA to the CPSAMPA format (a modification of XSAMPA) for use with CLEARPOND (Marian et al., 2012). The orthographic and phonological forms of each novel word were used to obtain average bigram and biphone probabilities in English, and English similarity was defined as a composite metric of *z*-transformed bigram and biphone probabilities.

To establish high and low English similarity thresholds, an English similarity percentile rank score was defined based on real English words. All five-letter English words in SUBTLEX-US (Brysbaert and New, 2009) with a frequency-per-million of 0.33 or greater were used to create the English similarity score. Each real word's score (i.e., average of *z*-transformed English bigram and biphone probabilities) was calculated and words were rank-ordered by English similarity. A High English similarity threshold was defined at the 20th percentile score, and 48 of the randomly generated novel words with scores above the threshold were selected for the first Familiar word list. A Low English similarity threshold was defined at the 99th percentile score, and 48 of the novel words with scores below the threshold were selected for the first Unfamiliar word list. Words in both lists were selected with the additional constraint of ensuring a balanced distribution of letters at word onset.

An additional 48 novel words in each condition (Familiar, Unfamiliar) were designed for use in the second session, which examined the effect of similarity to previously-learned words on new word learning. All new “terminus words” in the second session were substitution neighbors of a single item from that condition's “bridge word” list, learned in the first session. New terminus words were selected from a list comprising all non-word single-letter substitution neighbors of entries from the bridge word list (excluding duplicate entries, which were neighbors of multiple words in the bridge word list). In order to assess how well learners are able to utilize non-native patterns to learn other new words, English similarity was calculated for all generated entries and only new terminus words with scores below the Low English similarity threshold were selected for *both* the Familiar and Unfamiliar conditions. In other words, while the Familiar and Unfamiliar bridge word lists differed in English bigram/biphone probability for the first session, terminus words in the second session were equally dissimilar to English, thereby ensuring that effects of condition observed for terminus words could not be attributed to direct transfer from the L1. From this reduced list, 48 terminus words were randomly selected for each condition, with the constraints that each terminus word was a neighbor of a different word from the bridge word list and that the average English bigram/biphone probability did not differ between the second lists in each condition or between the bridge and terminus lists in the Unfamiliar condition (all *p*s > 0.1; see Supplementary Tables 1, 2 for bridge and terminus wordform statistics and stimuli).

All novel words were assigned a different English meaning for use during learning; the Familiar and Unfamiliar conditions both used the same list of 96 English words. To control for effects of individual novel-word—English-word pairings, two variants were created for each condition. The 96 English words were divided into A and B lists that each included equal numbers of concrete (e.g., “tree”) and abstract (e.g., “idea”) nouns (as determined by measures of imageability, see De Groot, 2006 for a similar approach). The two lists were matched for imageability, age of acquisition, and familiarity (Bristol norms) (Stadthagen-Gonzalez and Davis, 2006), as well as lexical frequency on the SUBTLEX-US zipf scale (Brysbaert and New, 2009; Van Heuven et al., 2014) (*p*s > 0.05; see Supplementary Tables 3, 4 for English words in lists A and B, as well as statistical comparisons of word characteristics in the two lists). For half of the participants in each language group, list A meanings were assigned to novel words in the bridge session and list B was used for the terminus session, while the other half of participants received list B meanings in the bridge session and list A meanings in the terminus session. In this way, each participant learned a translation of the same 96 English words (with each English word paired with a single novel word), with imageability, age of acquisition, familiarity, and lexical frequency controlled across the four list types (Familiar-Bridge, Familiar-Terminus, Unfamiliar-Bridge, Unfamiliar-Terminus). Lastly, in order to account for possible differences between groups in similarity between the novel wordforms and the wordforms of their direct English translations (e.g., a cognate effect or near-cognate effect), we confirmed that the number of novel word—English word pairs that had overlapping bigrams (e.g., a novel word “cohuz” paired with the English word “command”) did not differ between the Familiar and Unfamiliar Bridge word lists (2 and 0 out of 96, respectively) or between the Familiar and Unfamiliar Terminus word lists (1 out of 96 in both, *ps* > 0.05).

### Procedure

Participants learned the novel bridge and terminus word lists they were assigned over the course of two sessions spaced 2 weeks apart. In each session, each participant was given a sheet of paper containing all 48 novel bridge or terminus words and their meanings printed as paired associates (e.g., *haner*—bride). Participants were provided 16 min to silently learn as many words as they could, and were told that they would be tested immediately afterwards. While the use of a more structured task (e.g., timed presentation of individual word pairs) can be beneficial for isolating the mechanisms underlying effects on learning, the present study was designed to be an initial test of the hypothesis that similarity to native language words would modulate transfer of non-native knowledge. The use of carefully controlled word stimuli combined with a self-paced paired-associates task enabled us to assess the overall impact of native language similarity on non-native transfer without imposing constraints on learners' allocation of time to study individual words. This approach additionally allowed us to simultaneously test groups of participants in a classroom-like setting using a format commonly found in foreign language textbooks and study materials (see Prince, 1996; Laufer and Shmueli, 1997; Hermann, 2003; Webb, 2007 for similar approaches). The duration of the study phase was determined based on pilot data and prior studies utilizing similar paradigms (e.g., Pickering, 1982; Prince, 1996; Laufer and Shmueli, 1997; Webb, 2007). Following the study phase, participants were then given 6 min to write the matching novel word translations on a response sheet containing all 48 English meanings. The order of words was fixed across participants but randomized between learning and test. A research assistant later manually transcribed written responses onto a computer, which automatically scored participants' accuracy.

### Data Analysis

Response accuracy was calculated taking into account partially correct responses. Each correct letter in the correct position of a response scored 0.2 points, for a maximum score of 1. The effects of native and non-native word similarity on accuracy were analyzed with linear mixed effects-regression, using the lme4 package (Bates et al., 2014) in R (R Core Team, 2016). Models included fixed effects of Similarity Condition (Familiar, Unfamiliar) and Session (Bridge word, Terminus word), plus an interaction term. Imageability, familiarity, age of acquisition, and word frequency of the English translations were added as covariates. The models additionally included random intercepts for participants, word forms, and word meanings, allowing us to control for mean learning performance associated with individual participants and words. Models additionally included a by-participant random slope for Session and by-meaning random slopes for Session and Similarity (i.e., the “maximal” random effects structure^1^, Barr et al., 2013), allowing us to control for random variation in the fixed effects associated with individual participants and words. Significance of fixed effect estimates was evaluated using the Satterthwaite approximation for degrees of freedom. Follow-up comparisons on models' predicted marginal means (using Welch *t*-tests) also used the Satterthwaite approximation for degrees of freedom, and the Tukey correction for multiple comparisons.

## Results

**Figure 1:**
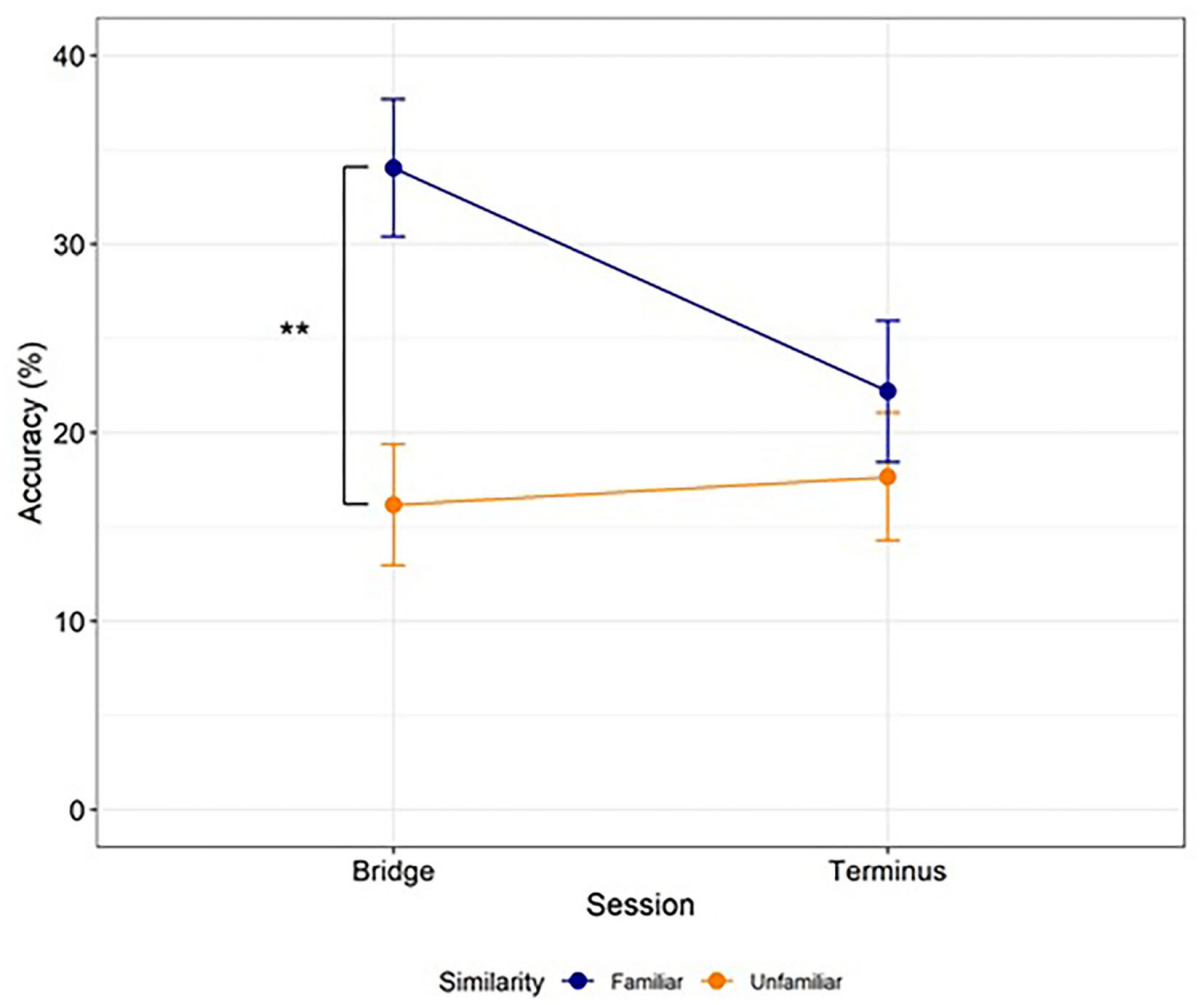
Word learning accuracy. Learners in the Familiar condition (blue) acquired more words in the Bridge session than learners in the Unfamiliar condition (orange), providing evidence of a between-language similarity benefit. Accuracy decreased from the Bridge to the Terminus session for learners in the Familiar condition. Dots and error bars represent observed values and standard error, respectively, by participants. Lines represent the best fit linear mixed-effects regression model. **p < 0.01.

We found a significant interaction between Similarity and Session [*Estimate* = −13.35, *SE* = 4.93, 95% CI (−23.01, −3.69), *t*(51.65) = −2.71, *p* = 0.009], as well as a main effect of Similarity [*Estimate* = 11.37, *SE* = 4.26, 95% CI (3.02, 19.72), *t*(43.98) = 2.67, *p* = 0.011] and a marginally significant main effect of Session [*Estimate* = −4.44, *SE* = 2.54, 95% CI (−9.42, 0.54), *t*(58.32) = −1.75, *p* = 0.086] (Figure 1). Follow-up comparisons on the model's predicted marginal means revealed that accuracy for the Familiar condition in the Bridge session *M* = 34.03, *SE* = 3.65, 95% CI (26.88, 41.19) was higher than for the Unfamiliar condition in the Bridge session *M* = 16.16, *SE* = 3.22, 95% CI (9.85, 22.48), *z* = −3.69, *p* = 0.001, and higher than accuracy for either the Familiar condition *M* = 22.18, *SE* = 3.74, 95% CI (14.85, 29.52), *z* = 3.2*, p* = 0.007 or the Unfamiliar condition in the Terminus session *M* = 17.66, *SE* = 3.38, 95% CI (11.03, 24.29), *z* = 3.26, *p* = 0.006. No other comparisons were significant.

The higher accuracy in the Bridge session for the Familiar condition compared to the Unfamiliar condition demonstrates a substantial benefit of native language similarity during self-directed vocabulary learning. However, the better learning observed for the Familiar condition did not carry through to the subsequent Terminus session, at which point there was no significant difference between word retrieval accuracy in the two groups.

The Terminus session contained entirely new vocabulary for participants to learn; all words were single letter substitution neighbors of words from the Bridge session (e.g., bridge word *haner* and terminus word *hajer*). To determine whether vocabulary that individuals learned in the Bridge session transferred to the Terminus session, we analyzed the data by first assigning each terminus word for each participant to one of three categories based on how well their substitution neighbors were learned during the Bridge session. Items in the Known Neighbor category were neighbors of bridge words that an individual got 4–5 out of 5 letters correct in the prior session. The Partly-Known Neighbor category included neighbors of bridge words with a score between 1 and 3 letters correct, and the Unknown Neighbor category included neighbors of bridge words that got a score of 0 letters correct. Note that items were assigned to Bridge-Knowledge conditions individually for each participant based on their performance in the Bridge session, and thus categories have an unbalanced number of items [see Table 1; χ^2^(2) = 96.39, *p* < 0.001].

**Table 1:** Percentage of terminus words with known, partly-known, and unknown neighbors.

	**Known neighbors**	**Partly-known neighbors**	**Unknown neighbors**
Familiar condition	29.2%	17.9%	53.1%
Unfamiliar condition	11.0%	17.4%	71.6%

*Percentages reflect the average proportion of Terminus words with Known, Partly-Known, and Unknown Bridge word neighbors in the Familiar and Unfamiliar conditions. Each Terminus word's (e.g., hajer) category was determined on a participant-by-participant basis depending on how well its orthographically-related Bridge word (e.g., haner) was learned in the previous test session. A Terminus word was categorized as having a Known Neighbor if the participant correctly recalled 4–5 out of 5 letters of its corresponding Bridge word, as Partly-Known if they correctly recalled 1–3 letters, and as Unknown if they correctly recalled 0 letters. To determine the overall distribution of Terminus words in each category, the percentage of words with Known, Partly-Known, and Unknown neighbors was calculated for each participant and then averaged across participants within the Familiar and Unfamiliar conditions*.

The model included fixed effects of Similarity Condition (Familiar vs. Unfamiliar) and Bridge-Knowledge (Known vs. Unknown Neighbor, and Known vs. Partly-Known Neighbor) plus interactions, as well as random intercepts for participant, word form, and word meaning, by-participant and by-form random slopes for Bridge-Knowledge, and by-meaning random slopes for Bridge-Knowledge and Similarity. Imageability, familiarity, age of acquisition, and word frequency of terminus words' English translations were entered as covariates.

**Figure 2:**
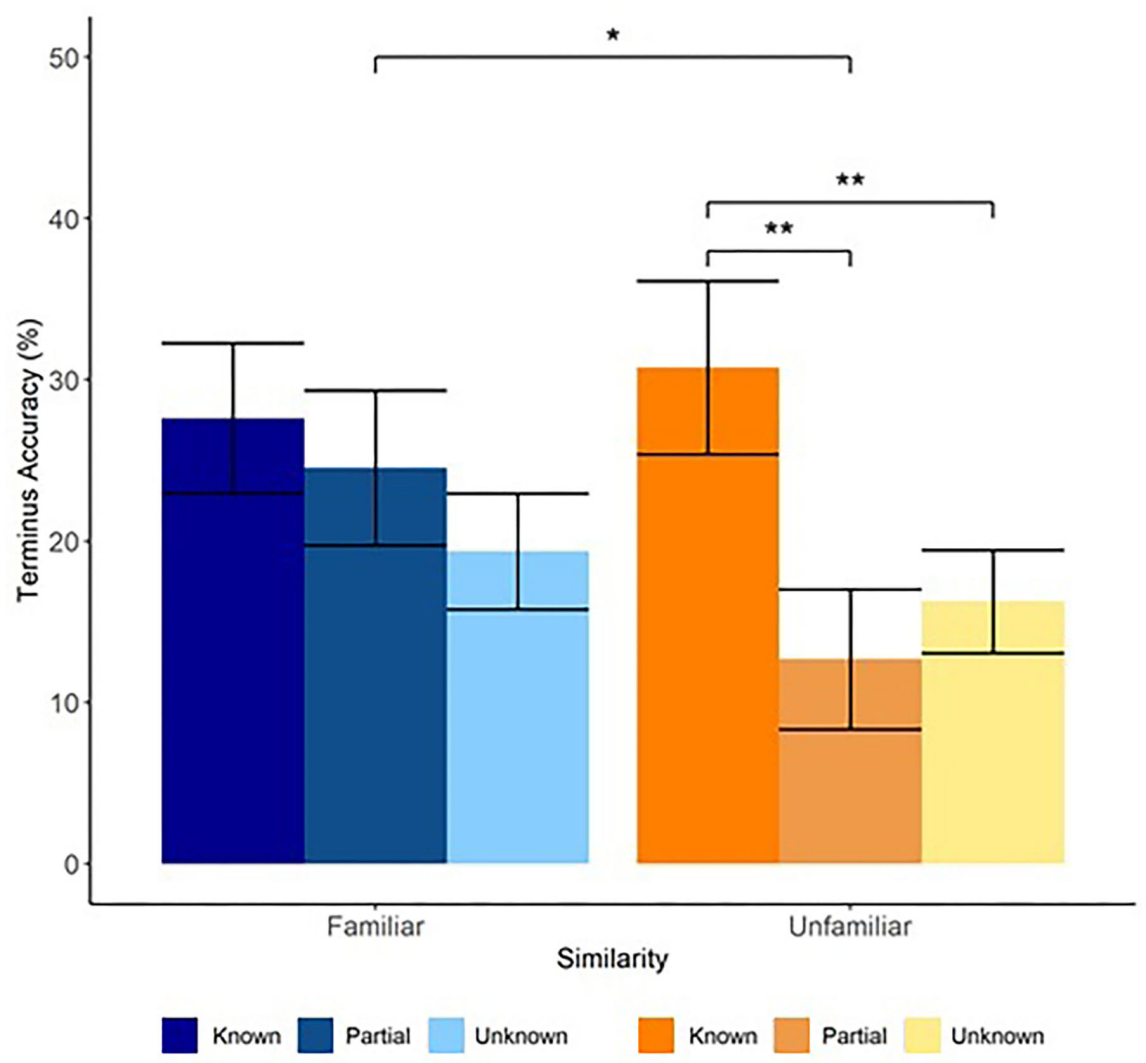
Similarity to previously-learned novel words influences later acquisition. Word learning in the Bridge session affected the likelihood of learning its orthographic neighbor in the Terminus session. The effect of prior novel word learning was moderated by similarity to the native language. Accuracy in the Unfamiliar condition was higher for Known Neighbor words (dark orange) than for Partly-Known Neighbor words (orange) and Unknown Neighbor words (light orange); accuracy for Partly-Known and Unknown Neighbor words did not differ from each other. Accuracy in the Familiar condition did not differ between Known Neighbor words (dark blue), Partly-Known Neighbor words (blue), and Unknown Neighbor words (light blue). Error bars represent standard error (by participants). *p < 0.05; **p < 0.01.

We found a significant interaction between Similarity and Bridge-Knowledge [Known vs. Partly-Known contrast, *Estimate* = 15.02, *SE* = 6.29, 95% CI (2.69, 27.34), *t*(66.5) = 2.39, *p* = 0.019], but not the [Known vs. Unknown contrast; *Estimate* = 6.25, *SE* = 5.25, 95% CI (−4.04, 16.54), *t*(96.6) = 1.19, *p* = 0.237] and a main effect of Bridge-Knowledge [Known vs. Unknown contrast, *Estimate* = −11.69, *SE* = 3.12, 95% CI (−17.82, −5.57), *t*(96.8) = −3.74, *p* < 0.001; Known vs. Partly-Known contrast, *Estimate* = −11.35, *SE* = 3.45, 95% CI (−18.11, −4.6), *t*(78.9) = −3.30, *p* =0.001] (Figure 2). Follow-up comparisons on the model's predicted marginal means revealed that accuracy for Known Neighbor words in the Unfamiliar condition *M* = 30.75 [*SE* = 5.37, 95% CI (20.0, 41.5)] was higher than for both Partly-Known Neighbors *M* = 12.68 [*SE* = 4.35, 95% CI (3.8, 21.5)], *t*(45.4) = 3.49, *p* = 0.003 or Unknown Neighbors *M* = 16.25 [*SE* = 3.2, 95% CI (9.8, 22.7)], *t*(50.0) = 3.18, *p* = 0.007. In contrast, accuracy for Known Neighbor words in the Familiar condition *M* = 27.60, [*SE* = 4.65, 95% CI (18.2, 37.0)] did not differ from either Partly-Known Neighbors *M* = 24.55, [*SE* = 4.81, 95% CI (14.8, 34.3)], *t*(24.4) = 0.67, *p* = 0.781 or Unknown Neighbors *M* = 19.36, [*SE* = 3.61, 95% CI (12.1, 26.7)], *t*(31.0) = 2.16, *p* = 0.094^2^. Partly-Known Neighbors did not differ from Unknown Neighbors in either condition. These results show that learning a word in the Bridge session increased one's chances of learning its neighbor in the Terminus session, providing evidence that similarity to previously-learned novel words benefits later vocabulary acquisition. Critically, similarity to previously-learned words influenced the types of words that people learned in the Unfamiliar condition more than in the Familiar condition. The significant difference between Known Neighbors and both Partly-Known and Unknown Neighbors, but not between Partly-Known and Unknown Neighbors further suggests that complete acquisition of a bridge word was necessary for participants in the Unfamiliar condition to benefit from similarity to previously-learned words. Partially learning a bridge word did not result in any differences between the Familiar and Unfamiliar conditions.

## Discussion

The goal of the present study was to determine how wordform similarity to the native language (as determined by bigram probability) influences acquisition of non-native vocabulary and sensitivity to non-native sublexical regularities. We found that although native language similarity provides short-term benefits, it can reduce reliance on non-native patterns during subsequent learning. Through continued use of an L2, the learner recognizes new patterns that determine how letters or sounds can combine to form words, and how words combine to form sentences. This process of extracting new patterns is also important for establishing continuous vocabulary learning, by ensuring that new words are accurately perceived and encoded in memory. Advanced L2 learners have been shown to benefit from L2 similarity during word learning (Stamer and Vitevitch, 2012; Ma et al., 2015), and in the current study, we found that similarity to other non-native words can also affect the earliest stages of vocabulary acquisition. Specifically, learning a word in the first session increased the likelihood that a similar word would be acquired in the subsequent session. Notably, while words that resembled the L1 were easier to learn at first, they had less of an influence on subsequent word learning. These results demonstrate the important roles of the native language, the burgeoning non-native vocabulary, and their interactions on new word learning.

Because of the way the new vocabulary in our study was designed, each word in the bridge session had a single substitution neighbor in the subsequent terminus session. These bridge-terminus word pairs allowed us to assess differences in word learning based on whether or not the learner already knew a similar word. Importantly, this is based not on intrinsic properties of the words, but instead on learners' idiosyncratic knowledge of patterns in the new word lists. Given the self-directed nature of the training session, the effect of similarity to previously-learned words that we observed may reflect how attention and study time were allocated to new words. Because overall accuracy did not improve between bridge and terminus sessions, the observed advantage for terminus words with already-acquired bridge neighbors comes at the expense of words with unlearned neighbors, consistent with prior self-directed word learning paradigms (Bardhan, 2010). The relative disadvantage for words with partly-learned neighbors may additionally result from the confusion that can occur between formally similar L2 words (e.g., the German words *Schafe*, meaning “sheep” and *schaffen*, meaning “create”; Laufer, 1988, 1989). Laufer (1988) conjectures that these types of “synform errors” may result from weak or unstable representations of L2 words in memory that could impair the learner's ability to distinguish between them or correctly map them to their corresponding meanings. Transfer from previously-learned wordforms may therefore have contrasting effects on subsequent learning depending on how well the initial words were learned, with facilitation from robustly encoded exemplars but interference from more unstable representations.

Notably, learners in the Familiar and Unfamiliar conditions differed in how much similarity to previously-learned words affected their continued learning. Even though bridge words in the first session were learned twice as well in the Familiar condition compared to the Unfamiliar condition, the effect of learning similar words in the terminus session was nearly twice as large for learners in the *Unfamiliar* condition. In the Unfamiliar condition, terminus words with learned bridge word neighbors were recalled with 2.65 times greater accuracy than words with unlearned bridge neighbors, compared to only a 1.65 times advantage in the Familiar condition. These terminus words in the second session were carefully designed to have equally low English similarity in both conditions, ensuring that this terminus word difference was due to effects of similarity to other non-native words, without confounding native and non-native word similarity. Together, these results indicate that although native language similarity provided an early benefit for word learning, it reduced the benefit of similarity to previously-learned non-native words in continued study.

Part of the task of learning a second language and achieving lexical competence involves building a foundation of L2 knowledge and a network of connections among L2 words and their meanings (Ellis and Beaton, 1993; De Groot, 2011), which can enhance the automaticity of L2 processing and minimize reliance on, and interference from, L1 knowledge (MacWhinney, 1997; Jiang, 2000). Connectionist models of bilingual language processing suggest that language selection and control can be accomplished over time via Hebbian learning and self-organizing representations that naturally cluster in language-specific ways due to greater feature overlap and co-activation of words within-languages, than across languages (e.g., Shook and Marian's, 2013 *BLINCS* model). Such a system could allow bilinguals to rely on bottom-up inputs, such as orthographic or phonological features, to selectively activate the appropriate language based on learned regularities within each language. For instance, language-specific sublexical cues, such as letter and bigram frequencies, can reduce the activation of cross-linguistic primes (Casaponsa and Duñabeitia, 2016; Dubey et al., 2018), and bilinguals can rely on language membership cues to guide lexical access (Grainger and Beauvillain, 1987; Vaid and Frenck-Mestre, 2002; Casaponsa et al., 2014) and speech production (Oganian et al., 2015). Participants in the current study who learned Familiar bridge words did not have orthographic cues that could reliably indicate language membership prior to lexical processing, which could have increased the activation of English representations relative to participants in the Unfamiliar condition. This may have stalled the process of linking new words into a coherent L2, interfering with transfer between the bridge and terminus words. In contrast, learners in the Unfamiliar condition were acquiring vocabulary that was unambiguously distinct from English. This distinction appears to be helpful in promoting extraction of non-native patterns to be used during new word learning.

The fact that the Familiar and Unfamiliar conditions did not differ in overall terminus word accuracy, however, may indicate that the two groups made use of different strategies or could have differed in other meaningful ways, such as in motivation, which has been shown to benefit word learning (Dörnyei and Ushioda, 2009; Dörnyei, 2019). For instance, the relative ease of learning bridge words that were similar to the L1 could have reduced motivation and effort in the Familiar condition, particularly during the second session when the task was unexpectedly more difficult. This could have elicited shallower processing of the terminus words during the word learning phase and consequently, reduced transfer from known bridge words. A complimentary interpretation would be that learners in the Unfamiliar condition benefited from the “desirable difficulties” (Bjork and Bjork, 2011) associated with learning more challenging bridge words. In language learning, retention in long term memory is generally improved when learning requires a greater depth of processing and involvement (Craik and Lockhart, 1972; Laufer and Hulstijn, 2001; Rice and Tokowicz, 2020), which can be instigated by material presented in a more difficult context (Schneider et al., 2002; Bjork and Kroll, 2015). Examples of desirable difficulties include repeated testing in place of passive study, or interleaving blocks of different word lists rather than blocked study (Schneider et al., 2002; Bjork and Kroll, 2015; Marecka et al., 2021). Our results suggest that difficulties caused by properties of the words themselves may also be targets for increasing long-term learning. Future research incorporating measures of motivation and/or manipulations of task engagement (e.g., through game-like formats, De Vos et al., 2019; Yang et al., 2020; see Derakhshan and Khatir, 2015 for review) could help elucidate the potential role of affective variables in determining the impact of cross-linguistic influence on transfer between non-native vocabulary.

In conclusion, we found that new vocabulary learning is affected by both similarity to one's native language and similarity to other newly learned words. Whereas native language similarity has a beneficial effect early on, it may decrease sensitivity to non-native word patterns that support later learning. This suggests that cross-linguistic influence is modulated by interactions between existing native and non-native word knowledge, and that initial similarity to the native language can have dynamically changing consequences over the course of novel word learning. This is because the words that one successfully learns early-on can influence the words that one acquires later, by driving attention toward new words that look more like already acquired ones. This suggests that cross-linguistic influences on initial vocabulary learning could potentially have cascading effects on the makeup of one's later vocabulary. Overall, these results demonstrate the complex relationship between native and non-native vocabulary, where similarity can have variable consequences for learning.

## Data Availability Statement

The raw data supporting the conclusions of this article will be made available by the authors, without undue reservation.

## Ethics Statement

The studies involving human participants were reviewed and approved by Northwestern University Institutional Review Board. The participants provided their written informed consent to participate in this study.

## Author Contributions

Conceptualization and design: VM, JB, and AB. Data collection: JB and AB. Statistical analysis: JB and SH. Writing—original draft preparation: VM and JB. Writing—review and editing: SH, VM, and AB. Project administration and funding acquisition: VM. All authors contributed to the article and approved the submitted version.

## Conflict of Interest

The authors declare that the research was conducted in the absence of any commercial or financial relationships that could be construed as a potential conflict of interest.

## Footnotes

^1^Note that no random slopes for Similarity were included for participants or word forms as each participant and word form was assigned to a single similarity condition (either Familiar or Unfamiliar). No random slope for Session was included for word form as each word form was presented in a single session (either bridge or terminus).

^2^Similar results were obtained when Bridge word accuracy was instead entered as a continuous variable, showing a significant effect of Bridge-Knowledge in the Unfamiliar condition [*Estimate* = 10.65, *SE* = 4.56, *t*(49.8) = 2.34, *p* = 0.024], but a marginal effect in the Familiar condition [*Estimate* = 7.71, *SE* = 3.95, *t*(83.8) = 1.95, *p* = 0.054].
