# Supplementary material for: Costs and Benefits of Native Language Similarity for Non-native Word Learning
Source: Front Psychol. 2021 May 28;12:651506. doi: 10.3389/fpsyg.2021.651506 (PMC8194348; doi:10.3389/fpsyg.2021.651506)
Supplement: Supplementary file 1 [file Table_1.DOCX]

Supplementary Material

| **Supplementary Table 1**. Bridge and Terminus Word Form Statistics | | | |
| --- | --- | --- | --- |
|  | Familiar | Unfamiliar | *p-value* |
| *Bridge* |  |  |  |
| Bigram Probability | 0.009 (0.003) | 0.002 (0.001) | <.001 |
| Orthographic Neighborhood Density | 0.56 (0.87) | 0.04 (0.2) | <.001 |
| Orthographic Neighborhood Frequency | 54.03 (295.27) | 0.11 (0.74) | 0.200 |
|  |  |  |  |
| *Terminus* |  |  |  |
| Bigram Probability | 0.004 (0.002) | 0.004 (0.002) | 0.54 |
| Orthographic Neighborhood Density | 0.14 (0.35) | 0.15 (0.36) | 0.94 |
| Orthographic Neighborhood Frequency | 21.5 (155.16) | 1.87 (7.04) | 0.383 |

*Note:* Bigram probability, neighborhood density, and neighborhood frequency were calculated based on similarity to English words using CLEARPOND (Marian, Bartolotti, Chabal, & Shook, 2012). Bigram probability reflects the average probability of each contiguous letter appearing after the other based on real English words (Medians: Familiar/Bridge = .008, Unfamiliar/Bridge = .001, Familiar/Terminus = .004, Unfamiliar/Terminus = .004). Orthographic neighborhood density reflects the number of English words that could be made through substitution, deletion, or addition of a letter (Median = 0 for all conditions). Orthographic neighborhood frequency reflects the average word frequency of orthographic neighbors (Median = 0 for all conditions).

| **Supplementary Table 2**. Bridge and Terminus Words | | | |
| --- | --- | --- | --- |
| Familiar | | Unfamiliar | |
| Bridge | Terminus | Bridge | Terminus |
| bafan | bafaz | biwop | bipop |
| bened | bezed | cihuz | cohuz |
| bogan | bogaj | dazuf | razuf |
| bonit | bohit | duhif | dusif |
| caruf | cahuf | febuz | fenuz |
| cobil | vobil | fohaf | fohal |
| cotam | zotam | gehaf | gehas |
| daben | dabev | gifob | gicob |
| ditel | dihel | huvuf | huvus |
| dolin | dowin | jezok | jezon |
| donop | dohop | jiwut | fiwut |
| feman | femab | jobav | cobav |
| furen | furun | jofup | jofuv |
| gedan | gedaf | kawob | fawob |
| gofen | gofev | kihab | kimab |
| haner | hajer | kuwez | kunez |
| hejen | hejef | lahoz | lahon |
| honak | hovak | lezaf | lesaf |
| horik | hozit* | lohuf | lomuf |
| jesan | jesaf | muhuw | muhul |
| jumon | jumoz | nahuz | sahuz |
| konud | kohud | nifab | nilab |
| korit | kowit | niwod | nirod |
| laret | lajet | nugov | nusov |
| luden | ludef | pihud | picud |
| mabon | mawon | piwuk | pimuk |
| melis | mulis | rijuf | dijuf |
| migen | migew | rujeg | ruzeg |
| mofen | mofej | rukow | sukow |
| naben | nabev | sohob | sohol |
| narer | naher | sojaz | sejaz |
| panid | pahid | tahob | lahob |
| pifan | pifaz | tiwoz | tinoz |
| posan | posaf | tugaz | tugar |
| remel | zemel | tuwop | guwop |
| rifan | rifab | vaduf | vadif |
| rucan | rucaw | vawub | cawub |
| saret | sajet | vobaf | tobaf |
| selim | sebim | vojug | vosug |
| tacen | tacun | wajud | cawub |
| tonop | tozop | wohif | wolif |
| tuson | tusof | wudov | wunov |
| vaben | vabew | wuheg | wuteg |
| vocon | vowon | wumof | wunov |
| warit | wahit | zagib | zagin |
| wehen | weheg | zibos | zinos |
| zolen | zolew | zodup | sodup |
| zutan | zutaf | zogir | zocir |

*One item in the Familiar Terminus list was excluded from analyses due to a substitution of two letters (rather than one) from its Bridge word.

| **Supplementary Table 3**. English Translation Statistics | | | |
| --- | --- | --- | --- |
|  | List A | List B | *p-value* |
| AoA (yrs) | 6.01 (1.57) | 6.11 (1.55) | 0.749 |
| Imageability | 454.9 (175.79) | 454.61 (183.91) | 0.994 |
| Familiarity | 529.78 (34.75) | 521.88 (34.78) | 0.269 |
| Log Frequency (zipf) | 4.25 (0.38) | 4.28 (0.36) | 0.683 |

*Note:* AoA, imageability (concreteness), and familiarity were determined based on the Bristol Norm database (Stadthagen-Gonzalez & Davis, 2006), and lexical frequency on the SUBTLEX-US zipf scale (Brysbaert & New, 2009; Van Heuven, Mandera, Keuleers, & Brysbaert, 2014).

| **Supplementary Table 4**. English Translation Words | |
| --- | --- |
| List A | List B |
| accent | airport |
| anthem | bacon |
| beard | bath |
| blouse | beach |
| bone | bride |
| bonus | coach |
| brand | comment |
| click | consent |
| cloud | contest |
| clue | crisis |
| code | cure |
| coin | desert |
| command | duty |
| concept | expert |
| culture | fate |
| debt | fist |
| event | fund |
| expense | ghost |
| favor | golf |
| flame | grass |
| gate | hammer |
| gold | idea |
| guilt | infant |
| guitar | iron |
| hint | litter |
| jail | mission |
| legend | muffin |
| lettuce | neck |
| load | network |
| moon | noise |
| nausea | noon |
| nurse | pill |
| option | pity |
| park | pony |
| percent | pride |
| rice | rainbow |
| river | request |
| score | rhythm |
| section | sheep |
| soup | shell |
| status | sink |
| stone | skill |
| string | soul |
| tooth | tale |
| torch | tension |
| tree | theme |
| vehicle | tongue |
| wheel | urge |
